# Asymptomatic arbovirus and campylobacter infections in German travelers to Asia

**DOI:** 10.1007/s00705-023-05870-y

**Published:** 2023-09-20

**Authors:** Werner Dammermann, Isabel-Elena Haller, Katrin Singethan, Christof D. Vinnemeier, Florian Hentschel

**Affiliations:** 1 Brandenburg Medical School (Theodor Fontane), Brandenburg, Germany; 2 University Medical Center Hamburg-Eppendorf, Hamburg, Germany; 3 Bundeswehr Institute of Microbiology, Munich, Germany; 4 Bernhard Nocht Institute for Tropical Medicine, Hamburg, Germany; 5 Zentrum für Innere Medizin II Hochschulklinikum Brandenburg der MHB, Hochstr. 29, 14770 Brandenburg an der Havel, Germany

**Keywords:** Dengue, Zika, Chikungunya, Hepatitis E, *Campylobacter jejuni*, ELISA

## Abstract

The true risk for many travel diseases is unknown because most studies do not detect asymptomatic infections. In this study, we performed ELISA for dengue virus (DENV), chikungunya virus (CHIKV), Zika virus (ZIKV), hepatitis E virus (HEV), and *Campylobacter jejuni* on samples from 81 healthy Germans before and after they traveled to Asia. ELISA found five seroconversions for *C. jejuni*, two for DENV, one for ZIKV, and zero for HEV. For CHIKV, three subjects were positive before travel and negative afterwards. None had symptoms. These infections would have gone unnoticed by retrospective studies. Therefore, the risk for these infections may be higher than previously estimated.

## Introduction

Over the last decades, worldwide travel has seen an exponential increase, with a peak of 1.3 billion tourist arrivals in 2017 [1]. Correspondingly, travel-associated infections have multiplied as well [2]. To assess the risk of such infections, most studies in this field retrospectively count spontaneously reported, symptomatic diseases in returning travelers [3, 4]. In contrast, little is known about the rate of asymptomatic infections in travelers [5].

We therefore longitudinally analyzed the sera of all individuals attending Hamburg-area travel health clinics before traveling to South or Southeast Asia. Blood samples were drawn before and after travelling, antibody titers for vector-borne diseases (VBDs) and food- and water-borne diseases (FWDs) endemic in that region were determined, and a questionnaire about clinical symptoms was filled out.

As examples of viruses causing VBDs, we chose dengue virus (DENV), chikungunya virus (CHIKV), and Zika virus (ZIKV) because they have recently caused several outbreaks, and they can be asymptomatic as well as highly symptomatic [6, 7]. Additionally, they can be detected easily using standardized commercial ELISA kits [8].

As examples of pathogens causing FWDs, we chose *C. jejuni* and hepatitis E virus (HEV) because they are endemic in Asia and can cause asymptomatic, mildly symptomatic, or highly symptomatic infections [9]. They also can be diagnosed using standard ELISA kits [10, 11].

## Materials and methods

Eighty-one consecutive travellers attending Hamburg travel health clinics from January 2017 to January 2018 were prospectively enrolled before and followed until after travelling to endemic countries in South and Southeast Asia (Table 1). None of the subjects reported previous infection with the investigated pathogens in the past.

**Table 1:** Characteristics of travelers, travel destinations and duration of stay. Asterisks (*) indicate countries with reported dengue outbreaks during the time of the investigation. Plusses (^++^) indicate countries with sporadic Zika infections during the time of investigation. All other pathogens showed baseline prevalence with no exceptional peaks.

Characteristic			
Subjects	Total		81
	Male		39 (48.2%)
	Female		42 (51.8%)
Age (years)	Total		34.2 ± 13.9
	Male		33.8 ± 13.2
	Female		34.9 ± 14.1
Travel duration	Days total		23.2 ± 11.0
Travel destinations	India*^,++^, Myanmar^++^, Thailand^++^, Cambodia^++^, Laos^++^, Nepal*, Vietnam*^,++^, Malaysia^++^, Indonesia^++^, Philippines^++^, Sri Lanka*		
Travel type	All-inclusive tours, backpacker tours		

During the time of the study, dengue outbreaks were reported in Nepal, Sri Lanka, and certain regions of India [12-13]. There was a baseline prevalence of Chikungunya, and there were some outbreaks, but all of them occurred in countries to which the subjects did not travel. Sporadic cases of endemic and imported Zika virus infections were seen in many Asian countries, but there were no outbreaks like the ones in South America during that time. Likewise, hepatitis E virus and *C. jejuni* were stably endemic, with no sudden peaks during 2017 [14, 15] (Table 1).

For each individual, a questionnaire was filled out, and antibody titers in serum were determined by ELISA. In brief, sera were screened for anti-HEV IgA, IgG, and IgM; DENV, ZIKV, and CHIKV IgG and IgM; and *C. jejuni* IgA and IgG according to the manufacturer’s instructions. The DENV test that we used had a sensitivity of > 94% for IgG and 38% for DENV IgM [16]. All of the test kits were obtained from Euroimmun AG, Lübeck, Germany). Statistical analysis was performed using GraphPad Prism (Graphpad Software Inc., version number 9.0).

## Results

Of the 81 individuals tested, none reported any clinical symptoms. Seven were seropositive for anti-DENV IgG before traveling, and nine after traveling (9% vs. 11%). One was seropositive for anti-DENV IgM before traveling, and one after traveling (1.2% vs. 1.2%). For anti-CHIKV IgG, five individuals were seropositive before traveling, and two after traveling (6.5% vs. 2.5%). For anti-CHIKV IgM, three were seropositive before traveling, and two after traveling (4% vs. 2.5%).

For anti-ZIKV IgG and IgM, none of the individuals were seropositive before traveling, and one was positive after traveling (0% vs. 1.2%). Fourteen individuals were seropositive for anti-*C. jejuni* IgG before traveling, and 19 after traveling (17% vs. 23.5%). Five were seropositive for anti-*C. jejuni* IgA before traveling, and four after traveling (6% vs. 4.9%). None were seropositive for anti-*C. jejuni* IgM before or after traveling (0% vs. 0%) (Table 2).

**Table 2:** Seroprevalence for chikungunya virus (CHIKV), dengue virus (DENV), and Zika virus (ZIKV) in absolute numbers and percentages. VBD, vector-borne disease

VBD seropositivity	CHIKV		DENV		ZIKV		All
	IgG	IgM	IgG	IgM	IgG	IgM	N
Pre-travel	5 (6%)	3 (4%)	7 (9%)	1 (1.2%)	0 (0%)	0 (0%)	81
Post-travel	2 (2.5%)	2 (2.5%)	9 (11%)	1 (1.2%)	1 (1.2%)	1 (1.2%)	

For anti-HEV IgG, nine individuals were seropositive before traveling, and nine after traveling (11% vs. 11%). For anti-HEV IgA, seven were positive before traveling, and six after traveling (9% vs. 7%). For anti-HEV IgM, none were positive before or after traveling (0% vs. 0%) (Table 3).

**Table 3:** Seroprevalence for hepatitis E virus (HEV) and *Campylobacter jejuni* in absolute numbers and percentages. FWD, food and water-borne disease

FWD seropositivity	HEV			*C. jejuni*		All
	IgA	IgG	IgM	IgA	IgG	N
Pre-travel	7 (9%)	9 (11%)	0 (0%)	5 (6%)	14 (17%)	81
Post-travel	6 (7%)	9 (11%)	0 (0%)	4 (4.9%)	19 (23.5%)	

## Discussion

To determine the risk for apparent as well as inapparent travel infections in a real-life scenario, we chose a consecutive, longitudinal, single-center serological approach.

For VBDs, we found one ZIKV and at least two DENV infections. The fact that we only found IgG but no IgM seroconversions for DENV could be due to the low sensitivity of the IgM test we used, so the real numbers might even be higher. We still preferred this test over the more sensitive ones because of its ability rule out cross-reactivity with CHIKV by performing parallel tests from the same manufacturer [17]. Regarding CHIKV, there was one individual who was IgM positive pre-travel and negative post-travel, which would theoretically suggest a symptomless infection at the beginning of the study from which the subject subsequently recovered. Moreover, three individuals were positive for anti-CHIKV IgG pre-travel and negative post-travel. Although anti-CHIKV IgG levels can decrease over time, it is unlikely that these three Germans had been infected in the past but all of them lost their seropositivity just while traveling to Asia [17]. We rather suspect the varying specificity of that test kit to be the cause [8].

Calculated incidences were 2% for DENV, 1.2% for ZIKV, and zero for CHIKV. This is slightly higher but still within the order of magnitude reported by others [6, 7].

Regarding FWDs, we found five seroconversions for anti-*C. jejuni* IgG, but none for IgA. This is not surprising, since the rise in IgA is usually transient and can be missed in a subclinical infection. No seroconversions for HEV were seen. The calculated incidence of 6.5% for *C. jejuni* was higher than expected. The incidence of HEV is, naturally, lower [9].

Of note, none of the individuals reported any symptoms, and even those who seroconverted, when specifically asked, did not recall being ill. This would mean that none of these infections would have been detected in a retrospective study or in a surveillance system relying on spontaneously reported infections [2, 55]. It would also be a possible explanation for some of the “autochthonous” VBD occurrences in European countries, where the primary case could not be identified [18, 19].

This small study has significant limitations. First, although we collected data on the time span traveled and the countries visited, some of the subjects provided only vague travel information such as “2017” or “India”. We know from other studies that there are significant regional and seasonal differences in the incidence and prevalence of these pathogens, especially for dengue virus [14, 20], and we were unable to determine whether these individuals were in the exact region of an outbreak at the time that it occurred. We also do not know the absolute number of people who travelled to a particular region because only individuals who spontaneously attended travel health clinics before departure were enrolled in the study. It is not clear if the study population was more or less at risk than those who did not seek advice. Finally, the low sensitivity of the DENV IgM test we used might have skewed the results [17], suggesting that the number of asymptomatic travel infections might actually have been higher than this study suggests.

## Abbreviations

*C. jejuni*: *Campylobacter jejuni*
CHIKV: Chikungunya virus
DENV: Dengue virus
ELISA: Enzyme-linked immunoassay
FWD: Food- and water-borne disease
HEV: Hepatitis E virus
Ig: Immunoglobulin
VBD: Vector-borne disease
ZIKV: Zika virus
